# Developmental Coordination Disorder Affects the Processing of Action-Related Verbs

**DOI:** 10.3389/fnhum.2016.00661

**Published:** 2017-01-10

**Authors:** Giovanni Mirabella, Sara Del Signore, Daniel Lakens, Roberto Averna, Roberta Penge, Flavia Capozzi

**Affiliations:** ^1^Department of Anatomy, Histology, Forensic Medicine and Orthopedics, University of Rome “Sapienza”Rome, Italy; ^2^IRCCS Neuromed HospitalPozzilli, Italy; ^3^Department of Pediatrics and Neuropsichiatry of Children and Adolescents, University of Rome “Sapienza”Rome, Italy; ^4^School of Innovation Sciences, Department of Industrial Engineering & Innovation Sciences, Eindhoven University of Technology, EindhovenNetherlands; ^5^IRCCS Children Hospital Bambino GesùRome, Italy

**Keywords:** developmental coordination disorder, embodied theory of language, action language, arm reaching movement, semantics

## Abstract

Processing action-language affects the planning and execution of motor acts, which suggests that the motor system might be involved in action-language understanding. However, this claim is hotly debated. For the first time, we compared the processing of action-verbs in children with Developmental Coordination Disorder (DCD), a disease that specifically affects the motor system, with children with a typical development (TD). We administered two versions of a go/no-go task in which verbs expressing either hand, foot or abstract actions were presented. We found that only when the semantic content of a verb has to be retrieved, TD children showed an increase in reaction times if the verb involved the same effector used to give the response. In contrast, DCD patients did not show any difference between verb categories irrespective of the task. These findings suggest that the pathological functioning of the motor system in individuals with DCD also affects language processing.

## Introduction

Traditionally, the neural circuits subserving language and actions were thought to be independent functional systems. This assumption has been challenged by embodied theories of language (e.g., Gallese and Lakoff, 2005; Meteyard et al., 2012). The central tenet of these theories is that semantic knowledge is, at least partially, grounded in sensory–motor systems. According to these theories, action language comprehension requires an internal re-enactment of the motor schemas associated with the presented word or sentence.

In spite of the remarkable amount of empirical data showing that action-related language affects the planning and execution of motor acts (e.g., Glenberg and Kaschak, 2002; Mirabella et al., 2012), there is no unambiguous demonstration that motor system activity is necessarily involved in the understanding of action-word meaning, as theories of embodied language would assume. In fact, even though there are strong indications that motor system activity can occur in association with action language comprehension (Hauk et al., 2004; Tettamanti et al., 2005), these findings could not unambiguously demonstrate whether or not the activation of the motor system is necessary for understanding action-word meaning (Mahon and Caramazza, 2008).

To address this hotly debated issue, we compared the performance of children with a typical development (TD) with that of children affected from Developmental Coordination Disorder (DCD) in two versions of a go/no-go paradigm in which verbs expressing either hand, foot or abstract actions were shown (Mirabella et al., 2012). In the so-called ‘semantic’ task participants are required to perform arm reaching movements toward a target when verbs expressing either hand or foot actions are shown, and to refrain from moving when abstract verbs are presented. In the color discrimination version, participants do not have to retrieve the semantic meaning of the verb to decide whether to move or not, but they have to respond based on the color of the printed verb. In a population of young adults, it was found that when the meaning of the verb (and not its color) was the cue for movement execution, both the reaction times (RT) and percentages of errors increased, provided that the verb described an action involving the same effector used to give the response (Mirabella et al., 2012; Spadacenta et al., 2014; but see also Sato et al., 2008). This ‘interference effect’ was speculatively interpreted as the result of an overexploitation of the regions of the motor system which control the movement of the effector employed, as these regions should be involved both in the organization of movement and in the comprehension of the action verb semantic.

In order to test this hypothesis, we assessed the role of the motor system in action language understanding by testing patients that are affected by DCD, a neurodevelopmental disorder characterized by diminished fine (e.g., manipulative skills such as using scissors, handwriting, buttoning shirts) and/or coarse motor coordination (e.g., clumsiness, inability to ride a bike). The motor skills deficit significantly and persistently interferes with activities of daily life and impacts academic performance, vocational activities, leisure, and play (Magalhaes et al., 2011). Crucially, these deficits are not explained by intellectual disability or visual impairment and are not attributable to a neurological disorder affecting movement (e.g., cerebral palsy). Increasing evidence indicates that in DCD patients motor networks are damaged (e.g., Zwicker et al., 2012; McLeod et al., 2014). In principle, these damages might account for deficits in imitation and motor imagery that are very often found in DCD patients (Gomez and Sirigu, 2015; Reynolds et al., 2015 for reviews). As both imitation and motor imagery deficits might be explained by a deficit in movement representation, i.e., a difficulty in creating internal models of motor acts (Wilson et al., 2004), it is plausible to hypothesize that if internal motor schemas are corrupted and they support action verb semantics, then also action language understanding should be impaired.

Our hypothesis is that if the motor system plays a role in action-verb processing, then DCD patients should exhibit a different modulation of the interference effect with respect to TD children.

## Materials and Methods

### Participants, Recruitment Details, and Assessments

From the outpatients of the Neurodevelopmental Disorders’s unit of the Department of Pediatric and Child Neuropsychiatry of Sapienza University of Rome, we selected 18 children diagnosed with DCD, (**Table 1** for all clinical data). The diagnosis of DCD was done on the basis of anamnestic and clinical data (family history, developmental and medical history, demographic data), neuromotor status and standardized assessments (Movement Assessment Battery for Children, M-ABC, Peabody picture vocabulary test, DCD-Questionnaire, Wechsler Intelligence Scale for Children) according to the diagnostic criteria of the Diagnostic and Statistical Manual, fifth edition (American Psychiatric Association, 2013) and those of the European Academy of Childhood Disability (Blank et al., 2012). Children with DCD were included if they met the following criteria: (i) a score of ≤15th percentile on the M-ABC or at the ≤5th percentile on one of the four M-ABC clusters (Henderson and Sudgen, 1992) which indicate a high level of motor impairment; (ii) a score in the range of ‘suspect’ or ‘indicative DCD’ on a parent-completed measure, the DCD-Questionnaire (Wilson et al., 2000, 2009); (iii) a score of ≥80 on the Wechsler Intelligence Scale for Children (Wechsler, 1991), which indicates a near-normal level of intellectual abilities; (iv) a score of ≥85 on the Peabody Picture Vocabulary-Test (Dunn and Dunn, 1981), which indicates a near-normal level of verbal comprehension, regardless of reading, or writing problems; (v) right-handedness as assessed by the Edinburgh handedness inventory. Children were excluded if they had comorbidity with other neurological (e.g., cerebral palsy) or psychiatric diseases (e.g., autism spectrum disorder, attention deficit hyperactivity disorder) that could explain their motor problems. Furthermore, we excluded children affected by specific learning and language disorders. All children were free of medication at study entry.

**Table 1:** Clinical data of DCD patients participating in the experiment.

	Sex	Age	DCD-Q total score	M-ABC total score	M-ABC manual dexterity	M-ABC ball skills	M-ABC static balance *plus* dynamic balance	WISC-III	Peabody PVT-test
1	M	8.7	57	18th	<5th	>15th	>15th	98	104
2	M	9.1	40	<1th	<5th	>5th and <15th	<5th	91	106
3	M	12.1	37	13th	>5th and <15th	>15th	>5th and <15th	129	127
4	M	10.3	38	6th	>15th	>5th and <15th	>5th and <15th	134	122
5	M	11.3	51	1th	<5th	>15th	<5th	80	111
6	M	10.6	42	<1th	>5th and <15th	<5th	>5th and <15th	147	108
7	M	8.2	46	15th	>5th and <15th	>5th and <15th	>15th	110	87
8	M	8.5	45	5th and 15th	<5th	>15th	>15th	100	102
9	M	10.2	49	16th	<5th	>15th	>15th	95	111
10	M	9.4	52	9th	>5th and <15th	>5th and <15th	>15th	107	94
11	M	9.6	48	16th	<5th	>15th	>15th	88	100
12	M	11.5	53	5th	<5th	>15th	>5th and <15th	121	117
13	M	8	38	<1th	<5th	>5th and <15th	>5th and <15th	89	110
14	M	10	35	<1th	>5th and <15th	>5th and <15th	<5th	114	102
15	M	7	46	4th	>5th and <15th	>5th and <15th	>15th	80	96
16	M	11.6	35	3th	>5th and <15th	>5th and <15th	>15th	89	107
17	F	9.6	49	8th	>15th	>15th	<5th	95	108
18	M	11.9	43	4th	>15th	>15th	<5th	118	116
Mean		9.87	44.7					104.7	107.1
*SD*		1.46	6.6					19.1	9.8


*For each patient sex, age, the total scores of DCD-Questionnaire (DCD-Q), the percentiles of the scores of the Movement Assessment Battery for Children (M-ABC) computed according to Henderson and Sudgen (1992), the scores of the Wechsler Intelligence Scale for Children (WISC-III), and the scores of the Peabody Picture Vocabulary-Test (PPVT) are given (see text for further details).*

As a control group, we recruited 18 right-handed children with TD without neurological disease and with normal or corrected-to-normal vision (16 males, 2 females, average age 10 years; *SD* = 0.7; range 7.5–11 years). The two groups did not have a significant different age (*t*-test *t*[24.9] = -0.35, *p* = 0.73). Unfortunately, we did not have the permission of collecting data on clinical scales, therefore it was not possible to determine whether TD children have subtle movement difficulties as measured either by the M-ABC or the DCD-Questionnaire.

All children were Caucasian, born in Italy, and attended the last 3 years of a primary school in Rome following regular education. All the families of both groups fell within a middle to upper-middle socioeconomic level (Hollingshead, 1975). This study was approved by the institutional review board of Sapienza University of Rome and was conducted in accordance with the ethical standards laid down in the Declaration of Helsinki of 1964. All parents provided written informed consent.

### Verbal Stimuli

In the semantic task, we selected 24 Italian verbs in the infinitive form (see **Table 2**), because in this form they engage only lexical–semantic retrieval processes, avoiding the syntactic and morphophonological integration processes that are engaged by the inflected forms. In the semantic task, (see below), eight verbs referred to arm/hand-related action (e.g., “tagliare,” “to chop”), eight referred to leg/foot-related action (e.g., “correre,” “to run”), and eight referred to abstract verbs (e.g., “scordare,” “to forget”). Verbs were matched for word length, syllables number, and total lexical frequency (Bertinetto et al., 2005). A one-way analysis of variance did not show significant differences between verb categories for word length [*F*(2,21) = 1.4, *p* = 0.27] syllables number [*F*(2,21) = 0.45, *p* = 0.64] or lexical frequency [*F*(2,21) = 0.6, *p* = 0.57]. Imageability of the verbs was measured on a 7-point scale, where 1 indicated that the verb could not be imagined while 7 indicated that the verb was very easy to imagine (Mirabella et al., 2012). A one-way analysis of variance of this feature of the verbs revealed a main effect (*F*[2,21] = 201.7, *p* < 0.001). *Post hoc* tests (pairwise comparisons with Bonferroni correction) showed that the imageability of arm/hand- and leg/foot-related verbs did not differ (all *p* < 1), but the imageability of movement verbs was higher than that of abstract verbs (all *p* < 0.0001).

**Table 2:** List of verbs used in the semantic and in the color discrimination tasks (items marked with a ‘x’).

	Verb	Letters	Syllables	Lexical frequency	Imageability	Translation
**Arm/Hand-related verbs**
	Firmare (x)	7	3	407	6.98	to sign
	Tagliare	8	3	379	6.95	to chop
	Disegnare (x)	9	4	190	6.93	to draw
	Applaudire	10	4	65	6.93	to applaud
	Timbrare (x)	8	3	8	6.86	to stamp
	Stappare	8	3	4	6.80	to uncap
	Svitare	7	3	3	6.84	to unscrew
	Sbottonare (x)	10	4	2	6.80	to unbutton

	Mean(± SEM)	8.4 ± 1.2	3.4 ± 0.5	132.3 ± 173.1	6.9 ± 0.1	

**Leg/Foot-related verbs**
	Correre	7	3	662	6.95	to run
	Camminare (x)	9	4	234	6.98	to walk
	Marciare (x)	8	3	45	6.68	to march
	Pedalare (x)	8	4	37	6.89	to pedal
	Zoppicare	9	4	10	6.55	to hobble
	Calciare	8	3	8	6.93	to kick
	Saltellare (x)	10	4	6	6.95	to jump
	Pattinare	9	4	4	6.75	to skate

	Mean(± SEM)	8.5 ± 0.9	3.6 ± 0.5	125.8 ± 229.9	6.8 ± 0.2	

**Abstract-related verbs**
	Amare	5	3	818	5.64	to love
	Temere (x)	6	3	334	5.25	to fear
	Approvare	9	4	254	5.68	to approve
	Meditare (x)	8	4	34	5.45	to meditate
	Sopportare (x)	10	4	154	5.55	to bear
	Odiare	6	3	115	5.11	to hate
	Ammirare	8	4	110	5.61	to admire
	Scordare (x)	8	3	42	5.16	to forget

	Mean(± SEM)	7.5 ± 1.7	3.5 ± 0.5	232.6 ± 275.5	5.4 ± 0.2	


*For each item, number of letters, number of syllables, lexical frequency, imageability and English translation are given. Mean number of letters, syllables, lexical frequency and imageability (±SD) are reported separately for each verb category.*

In the color discrimination task, (see below), half of the verbs (four for each category) were selected from the list used in the semantic task, and were matched for syllable number, word length and lexical frequency. A one-way ANOVA did not show significant differences between verb categories for syllables number [*F*(2,10) = 0.45, *p* = 0.65], word length [*F*(2,10) = 0.49, *p* = 0.62], or lexical frequency [*F*(2,10) = 0.49, *p* = 0.62]. Again, a one-way ANOVA on imageability ratings showed a main effect [*F*(2,10) = 174.9, *p* < 0.0001], due to lower imageability ratings for abstract verbs than the other two categories (pairwise comparisons with Bonferroni correction, all *p* < 0.0001), while the imageability of arm/hand- and leg/foot-related verbs did not differ (all *p* < 1). We used only half of the verbs in each category to reduce the overall number of trials.

Due to a technical mistake a version of the semantic task and color discrimination task with, respectively, 30 and 15 verbs was administered to five DCD and six TD children. These versions were identical to those employed in Mirabella et al. (2012). As the results did not differ, in the following we averaged the two data sets, with the exception of the item by item analysis where only verbs that were presented in all subjects were considered.

### Experimental Apparatus

**FIGURE 1:**
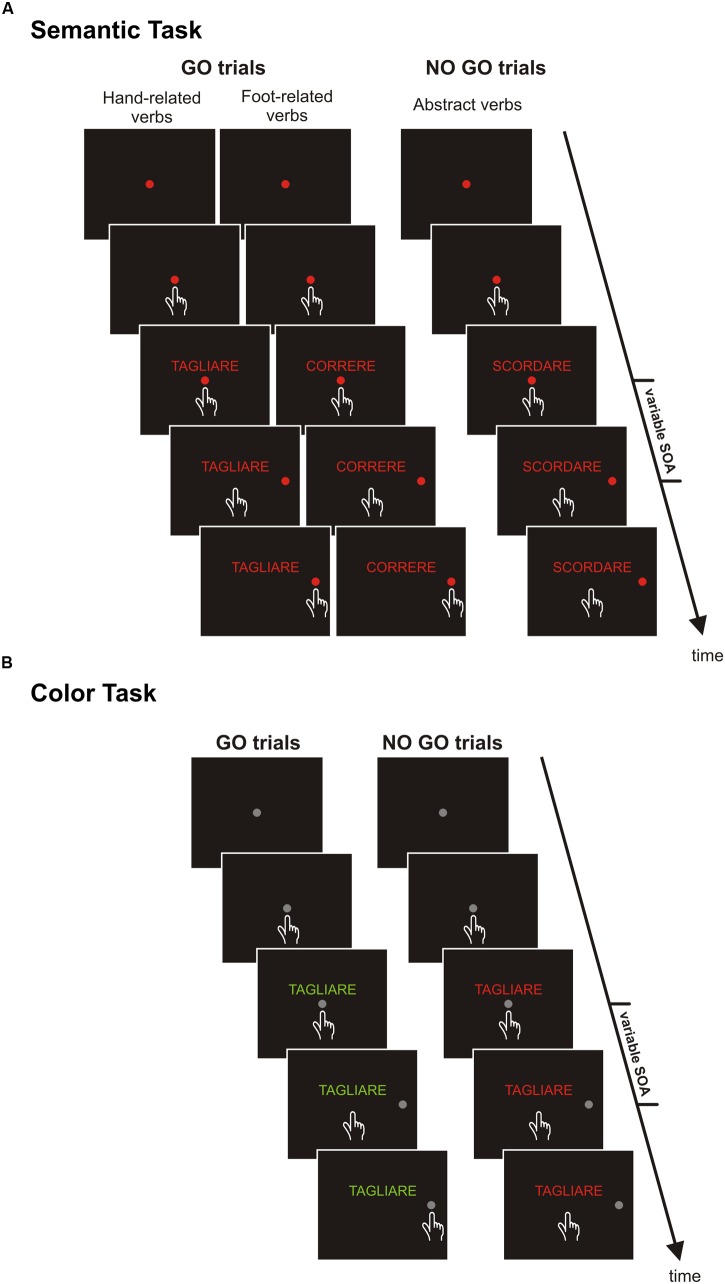
**Schematic representations of experimental tasks.** **(A)** Semantic task. Each trial started with the presentation of a central red circle that subjects were instructed to touch and hold for 400–800 ms. Thereafter, a verb was shown just above the central stimulus. After one of two possible delays (short or long SOA, 53.2 and 332.5 ms, respectively) a peripheral target appeared. Participants were required to touch it, when the meaning of the verb referred to concrete actions (go-trials), or to refrain from moving, when the meaning of the verb referred to abstract actions (no go trials). **(B)** Color-discrimination task. The overall structure and the sequence of events were the same of the semantic task, except for two exceptions. Both the central and the peripheral stimuli were gray, and participants were required to reach the target when verbs were printed in green (go-trials) and to keep the index on the central position when verbs were printed in red (no go trials).

Subjects were seated in a darkened and silent room, in front of a 17-inch PC monitor (CRT non-interlaced, refresh rate 75 Hz, 640 × 480 resolution, 32-bit color depth) on which visual stimuli were presented, (see below for their description), against a dark background of uniform luminance (<0.01 cd/m^2^). The PC monitor was equipped with a touch screen (MicroTouch; sampling rate 200 Hz) for touch-position monitoring. A free, non-commercial software package (CORTEX, developed at the National Institutes of Health, Bethesda, MD, USA), was used to control stimulus presentation and to collect behavioral responses. The temporal arrangements of stimulus presentation were synchronized with the monitor update rate. Participants performed, in separate sessions counterbalanced across participants, two tasks (**Figure 1**): (i) the semantic task, and (ii) the color discrimination task.

### Semantic Task

Each trial began with the presentation of a central red circle (diameter: 3.2 degrees of visual angle [dva], or 2.8 cm) that participants had to touch with their index finger and to hold (continue touching) for a variable period (400–700 ms). Thereafter, a verb was presented just above the central circle and participants were instructed to carefully read it. When the verb referred to a concrete action (go trials, frequency: 66% of times) participants had to reach and hold for a variable period (300–400 ms) a peripheral red circle (3.2 dva or 2.8 cm diameter) appearing to the right of the screen at an eccentricity of 9.1 dva (or 8 cm). Conversely, when the verb described an abstract action (no-go trials, frequency: 33% of times) participants had to keep the index finger still on the central stimulus for 400–800 ms (**Figure 1A**). Therefore, participants had to move on the basis of a semantic judgment. Successful trials were signaled by acoustic feedback. The go-signal, given by the presentation of the peripheral target, was delivered either 53.2 ms, (i.e., four refresh rates, RRs), after the presentation of the verb or at a stimulus onset asynchrony (SOA) of 332.5 ms (i.e., 25 RRs). The purpose of using these two SOAs was to obtain data comparable with those of our earlier studies (Mirabella et al., 2012; Spadacenta et al., 2014). Originally, we used these two SOAs because they gave two time points around the time window within which Sato et al. (2008) found that RTs increased whenever the action expressed by the verb involved the same effector used to give the motor response, i.e., 150 ms after the presentation of the verb.

Verbs were printed in red and remained visible until the end of the trial. Each verb was presented until 10 correct responses were given for each SOA; thus the experiment consisted of 480 correct trials, run in three blocks. Error trials were repeated until participants completed the entire block. All experimental conditions (verbs and SOAs) were randomized. To discourage participants from slowing down during the task, we set an upper reaction time limit for go-trials: every time RTs were longer than 600 ms, go-trials were signaled as errors and aborted when the participant left the touch form the central stimulus (overtime reaching-trials). Overtime reaching-trials were included in the analyses to avoid to cut the right tail of the RT distribution, and they accounted for 8.6% of the total trials. Resting periods were allowed between blocks whenever requested.

### Color Discrimination Task

In contrast to the semantic task, in the color discrimination task participants were instructed to execute or refrain from executing the movement according to the color in which verbs were printed (**Figure 1B**). Each trial started with the presentation of a central target (a gray circle with a diameter of 3.2 dva or 2.8 cm) that participants had to touch and hold for a variable period (400–700 ms). Thereafter, a verb was displayed above the central target. When the verb was printed in green, subjects were instructed to reach, as fast as possible, the peripheral target (a gray circle with a diameter of 3.2 dva or 2.8 cm) that was presented on the right side with an eccentricity of 9.1 dva (or 8 cm). Conversely, when the verb was printed in red participants had to refrain from moving. As in the semantic task, we set an upper RT limit for go-trials to 600 ms. Overtime reaching-trials were again included in the analyses, and accounted for 9.7% of the total trials. In total four verbs for each category were used (**Table 2**). Each verb was presented until 20 correct responses were given for each SOA; half of the time it was printed in green and the participant had to move (go trials, frequency: 50% of times) and half of the time it was printed in red and the participant had to stop (no-go trials, frequency: 50% of times). Thus, the experiment consisted of 480 correct trials, run in two blocks. As in the previous task, all experimental conditions (verbs and SOAs) were randomized and resting periods were allowed between blocks if requested. We did not employ all the eight verbs for time constrains.

### Data Analyses

For each participant, the mean RTs, the mean movement times (MTs) of correct trials, and the mean error rates were calculated for each verb category. RTs were determined as the time difference between time of the occurrence of the go-signal and movement onset. MTs were computed as the time difference between time of movement onset and the time at which subjects touched the peripheral target. For each participant, those trials in which the RTs were lower than 80 ms were eliminated, as they were considered premature responses. In addition, trials with RTs longer than the mean plus three SDs and shorter than the mean minus three SDs were excluded from the analysis. In total 2.2% of the data were discarded. We defined errors as those instances in which participants remained still on the central stimulus, instead of reaching the peripheral target. We did not consider: (i) errors on no-go trials (because in the semantic task they were related to verbs whose meaning did not clearly involve movement of the effectors); (ii) early responses, i.e., instances in which participants touched the monitor before the appearance of the central stimulus or instances in which they moved the arm while holding the central stimulus; (iii) missed responses, i.e., instances in which participants did not touch the central target at the beginning of the trial or they never moved their finger from it. Both missed and early responses can be taken as indices of the attention that a given participant pays to the task. As expected, these kind of errors were more frequent in DCD than in TD children, being 16 and 5% of the total number of trials, respectively. Crucially, in each group, the percentage of these mistakes did not differ across the semantic and color discrimination tasks. ANOVAs were performed to analyze RT differences and errors across conditions. Bonferroni corrections were applied for all multiple comparisons, and across all exploratory 2X2X2 ANOVA’s, we used an α of 0.05/7 = 0.007.

We computed the partial omega-squared (ω_p_^2^) as effect sizes for each ANOVA, with values of 0.139, 0.058, and 0.01 indicating large, medium, and small effects, respectively, and Hedges’ *g* as the effect size for *t*-tests with values of 0.2, 0.5, and 0.8 indicating large, medium, and small effects (Lakens, 2013). For dependent *t*-tests, we provide correlations (*r*) between measurements (in addition to means and standard deviations). We computed the ω_p_^2^ and the Hedges’ *g* because they provide a measure of the effect of a given manipulation regardless of other factors that have been manipulated, allowing a comparison with related studies. All data, including analysis scripts in SPSS and R, can be downloaded from https://osf.io/an5f7/?view\_only=b2c6a1a5169b4b5d9f605a24ac59407e.

## Results

### Semantic Task: Reaction Times

**FIGURE 2:**
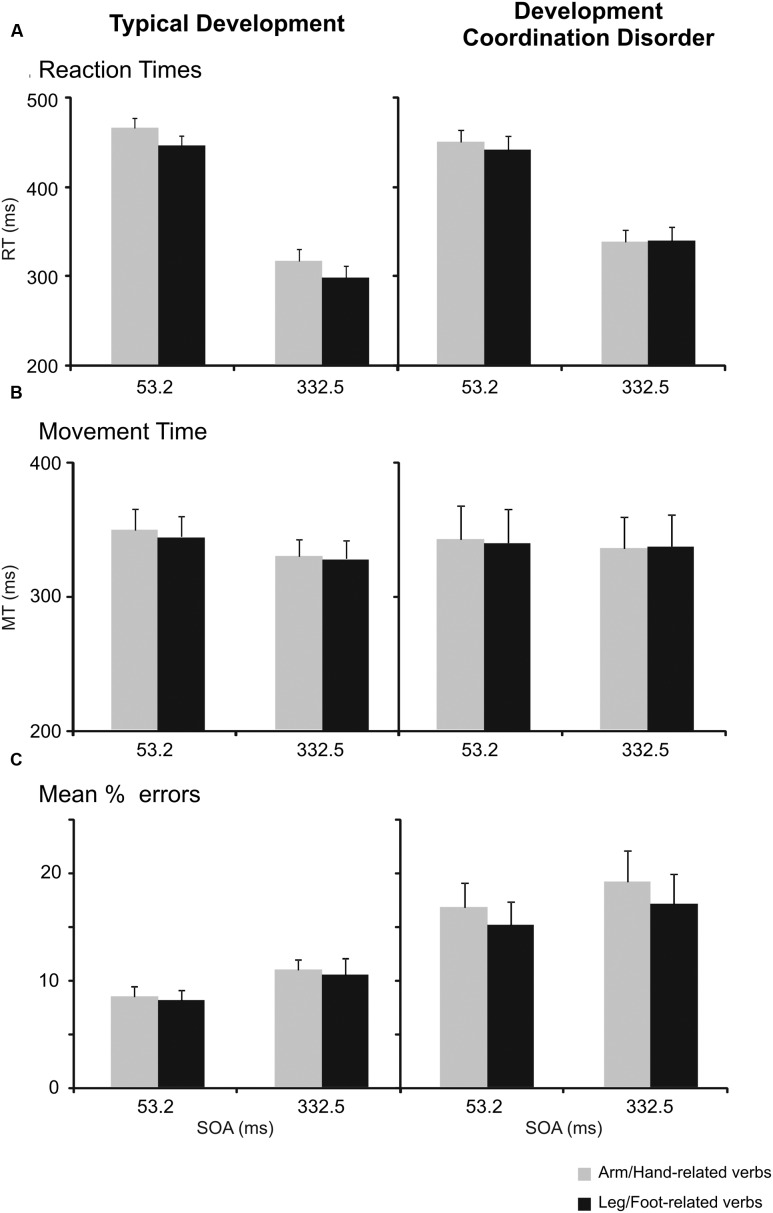
**Interference effect in the semantic task in children with typical development (TD) and in those affected by developmental coordination disorder (DCD).** **(A)** Mean values of reaction times (RTs) of arm reaching movements to arm/hand- and leg/foot-related verbs at the a stimulus onset asynchrony (SOA) of 53.2 ms and at an SOA of 332.5 ms. Bars represent the SEM. **(B)** Mean values of movement times (MTs). Bars represent the SEM. **(C)** Mean percentage of errors, i.e., frequency of times in which participants did not move toward the peripheral target when they had to. Bars represent the SEM.

To assess the effect of verb processing on the RTs of reaching arm movements, (see **Table 3** and **Figure 2A**), we performed a three-way repeated-measures ANOVA [between-subjects factor: Group (DCD, TD); within-subjects factor: verb category (arm/hand-related, leg/foot-related) and SOA (short [53.2 ms], long [332.5 ms]).

**Table 3:** Summary of behavioral measurements for children affected by developmental coordination disorder (DCD) and for those with typical development (TD).

	DCD children				TD children			
		
	Semantic task		Color discrimination task		Semantic task		Color discrimination task	
				
	Short SOA	Long SOA	Short SOA	Long SOA	Short SOA	Long SOA	Short SOA	Long SOA
RTs arm/hand related verbs	444.9 (58)	332.1 (67.6)	430.3 (47.2)	282.7 (59.8)	464.4 (45.1)	316.0 (52.7)	411.3 (34.1)	273.2 (42.9)
RTs leg/foot related verbs	437.3 (55.9)	333.1 (67.5)	430.6 (45.5)	279.2 (69.6)	444.6 (44.9)	297.5 (55.7)	414.6 (39.8)	263.4 (28.0)
RTs abstract verbs	–	–	424.9 (43.5)	276.5 (59.5)	–	–	424.8 (35.7)	284.7 (40.6)
MTs arm/hand related verbs	341.7 (105.2)	334.8 (99.1)	325.9 (83.6)	340.9 (85.3)	347.9 (68.8)	328.3 (55.5)	345.3 (69.2)	353.2 (59.4)
MTs leg/foot related verbs	339.7 (105.4)	336.5 (99.5)	325.5 (83.4)	338.4 (79.9)	343.9 (62.3)	326.8 (57.9)	355.8 (64.8)	355.1 (68.3)
MTs abstract verbs	–	–	334.0 (85.2)	331.6 (78.4)	–	–	343.3 (71.9)	354.8 (66.0)
Mean % errors arm/hand related verbs	16.7 (9.8)	19.1 (12.5)	11.6 (8.3)	13.7 (11.1)	8.5 (3.9)	10.9 (7.5)	10.3 (7.4)	12.2 (9.1)
Mean % errors leg/foot related verbs	15.0 (9.1)	17.0 (11.7)	12.1 (8.7)	14.6 (8.6)	8.1 (4.1)	10.5 (6.6)	11.4 (8.9)	10.0 (8.3)
Mean % errors abstract verbs	–	–	11.2 (7.7)	12.6 (9.5)	–	–	10.9 (7.5)	10.9 (7.4)


*In all cases the average values of reaction times (RTs), movement times (MTs), mean percentage of errors, (i.e., frequency of times in which participants did not move toward the peripheral target when they had to), and the corresponding SDs are reported.*

We found a main effect of SOA indicating that participants responded slower during trials with a short SOA (*M* = 448, *SD* = 50) than during trials with a long SOA (*M* = 320, *SD* = 61), *F*(1,34) = 237.09, *p* < 0.001, ω_p_^2^ = 0.87, mean difference (*M*_diff_) = 128, 95% confidence interval (CI) [110; 146]. Furthermore, the factor verb category showed a main effect {*F*(1,34) = 21.18, *p* < 0.001, ω_p_^2^ = 0.36, *M*_diff_ = 11, 95% CI [6; 17]}. Indicating that, overall, participants responded faster to foot related verbs (*M* = 378, *SD* = 49) than to hand related verbs (*M* = 389, *SD* = 50). This effect is qualified by an interaction between group and verb type, *F*(1,34) = 10.36, *p* = 0.003, ω_p_^2^ = 0.21, 95% CI [0.004; 0.42]. In fact, while TD children showed the expected interference effect, i.e., they had significantly slower RTs (*M* = 390, *SD* = 44) when they responded to arm/hand-related verbs than to leg/foot-related verbs (*M* = 371, *SD* = 44, *r* = 0.953), *t*(17) = 5.98, *p* < 0.001, Hedges’ *g* = 0.43, *M*_diff_ = 19, 95% CI [12; 26], DCD children did not show a significant difference between the RTs to arm/hand (*M* = 389, *SD* = 56) and leg/foot-related (*M* = 385, *SD* = 55, *r* = 0.960) verbs, *t*(17) = 0.91, *p* = 0.374, Hedges’ *g* = 0.07, *M*_diff_ = 3, 95% CI [-4; 11]. No other effects were statistically significant after the Bonferroni correction.

Because the lack of a significant effect for DCD children does not imply the lack of an effect, we tested for equivalence using the two one-sided tests procedure (Schuirmann, 1987). A power analysis for a dependent *t*-test indicated our sample size provided 80% power to observe equivalence with an equivalence range of *d*_z_ = -0.69 to 0.69, given the sample size of 18 participants in each between subject condition. Thus, we tested whether the difference between RTs for FOOT and ARM related words was statistically smaller than *d*_z_ = 0.69 and larger than *d*_z_ = -0.69, which was the case, *p* = 0.03. A default Bayesian *t*-test further supported the idea that the data provided evidence for the null model, BF_01_ = 0.35.

**FIGURE 3:**
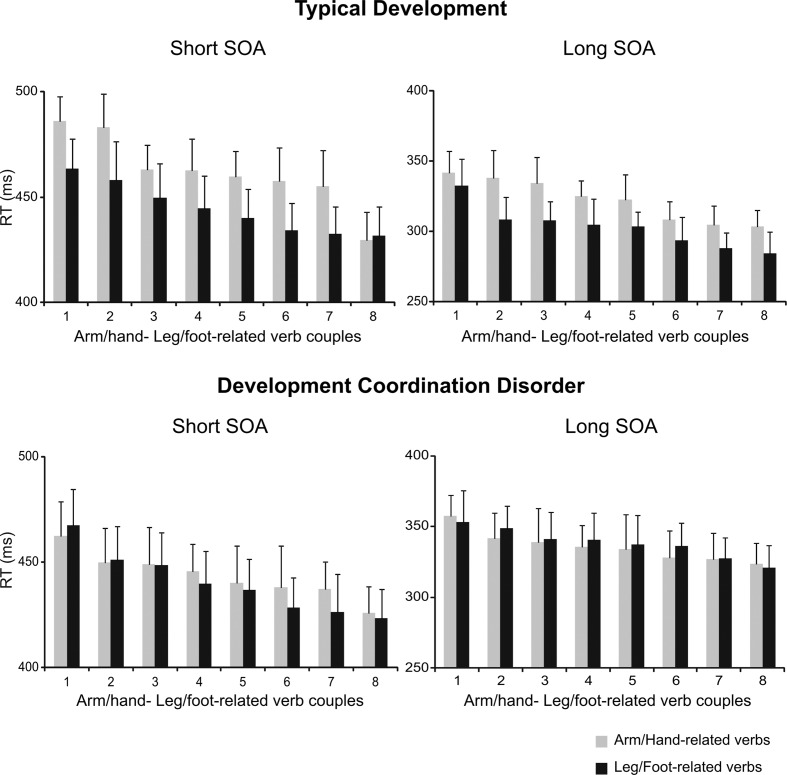
**Interference effect in the semantic task at single verb level in children with TD and in those affected by DCD.** Ranking of the mean RTs for each arm/hand- and foot/leg-related verb at a SOA of 53.2 ms (short SOA) and at an SOA of 332.5 m (long SOA) for TD and DCD children. Mean RTs of arm/hand- and foot/leg-related verb are paired according to their ranking status (i.e., the longest mean RT for an arm/hand-related verb is paired with the longest mean RT for a foot/leg-related verb and so on).

Importantly, the modulation of RTs linked to verb categories was present for each individual verb and at both SOAs only in TD children (**Figure 3** upper panels), but not in DCD patients (**Figures 3** lower panels), suggesting that this phenomenon is not restricted to a few verbs, but is consistent across all chosen items.

### Semantic Task: Movement Times

The same analyses on MTs (**Table 3** and **Figure 2B**) revealed a main effect of SOA. Participants moved more slowly during trials with a short SOA (*M* = 343, *SD* = 86) than during trials with a long SOA (*M* = 332, *SD* = 79), *F*(1,34) = 11.94, *p* = 0.001, ω_p_^2^ = 0.23, *M*_diff_ = 12, 95% CI [4; 19]. The interaction between SOA and Group was not significant, {*F*(1,34) = 4.09, *p* = 0.051, ω_p_^2^ = 0.08, 95% CI [-0.03; 0.29]}.

### Semantic Task: Errors

A three-way repeated-measures ANOVA [between-subjects factor: Group (DCD, TD); within-subjects factor: verb category (arm/hand-related, leg/foot-related) and SOA (short [53.2 ms], long [332.5 ms])] of the errors in the semantic task (**Table 3** and **Figure 2C**) revealed a main effect of group and of SOA. The main effect of group was due to higher error rates in DCD children (*M* = 16.96, *SD* = 10.09) compared to TD children (*M* = 9.52, *SD* = 4.86), *F*(1,34) = 7.95, *p* = 0.008, ω_p_^2^ = 0.16, *M*_diff_ = 7.44, 95% CI [2.08; 12.80]. The main effect of SOA was due to the fact that there were more errors in long SOA (*M* = 11.99, *SD* = 6.57) than in short SOA (*M* = 14.51, *SD* = 8.83), *F*(1,34) = 6.99, *p* = 0.012, ω_p_^2^ = 0.14, *M*_diff_ = 2.31, 95% CI [0.56; 4.06]. There was no interaction between Group and Verb, *F*(1,34) = 1.32, *p* = 0.26, ω_p_^2^ = 0.01, 95% CI [-0.03; 0.22].

### Color Discrimination Task: Reaction Times

**FIGURE 4:**
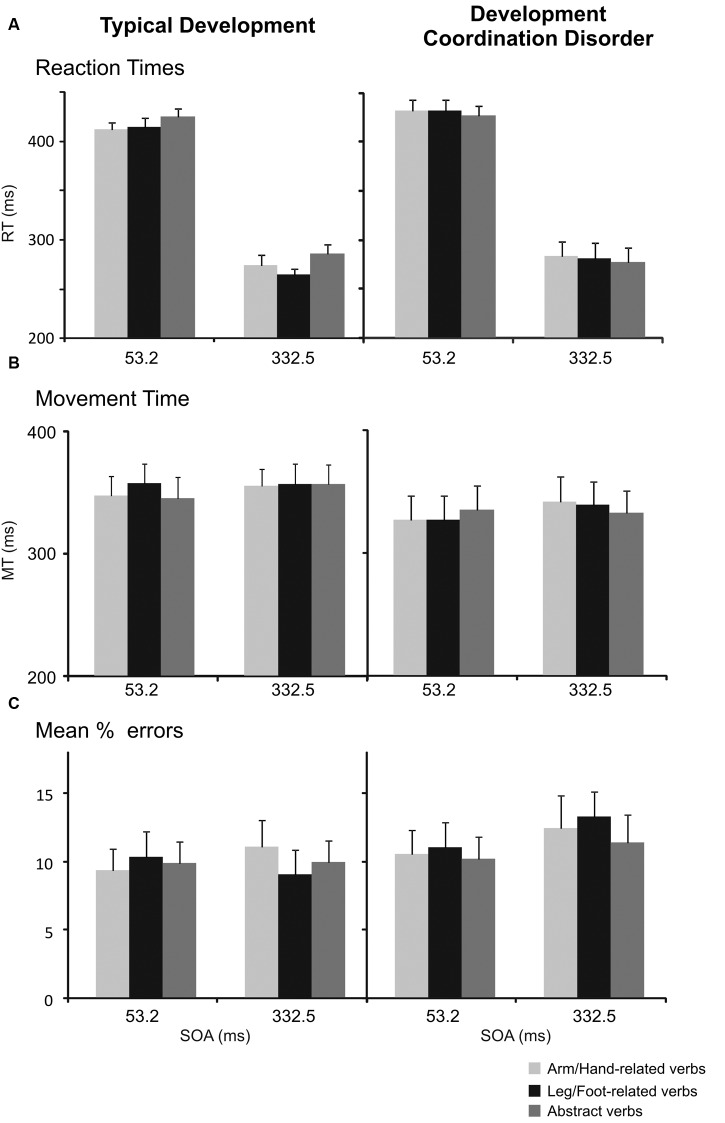
**Interference effect in the color discrimination task in children with TD and in those affected by DCD.** **(A)** Mean values of RTs of arm reaching movements to arm/hand-, leg/foot-, and abstract-related verbs at the SOA of 53.2 ms and at an SOA of 332.5 ms. Bars represent the SEM. **(B)** Mean values of MTs. Bars represent the SEM. **(C)** Mean percentage of errors, i.e., frequency of times in which participants did not move toward the peripheral target when they had to. Bars represent the SEM.

A three-way repeated-measures ANOVA [between-subjects factor: Group (DCD, TD); within-subjects factor: verb category (arm/hand-related, leg/foot-related, abstract-related) and SOA (short [53.2 ms], long [332.5 ms]) of the RTs in the color discrimination task revealed a main effect of SOA, with slower responses on short SOA trials (*M* = 423, *SD* = 29) than on long SOA trials (*M* = 277, *SD* = 49), *F*(1,34) = 553.52, *p* < 0.001, ω_p_^2^ = 0.94, *M*_diff_ = 146, 95% CI [134; 159]. The interaction between Group and Verb was significant, *F*(1,68) = 6.81, *p* = 0.002, ω_p_^2^ = 0.14, 95% CI [0.00; 0.29], caused by faster RT on foot-related words (*M* = 339, *SD* = 28) than abstract related words (*M* = 355, *SD* = 32, *r* = 0.79) in TD children, *t*(17) = 3.40, *p* = 0.003, Hedges’ *g* = 0.50, *M*_diff_ = 16, 95% CI [6; 26]. Differently, DCD children did not show significant differences between the RTs to arm/hand-related verbs (*M* = 357, *SD* = 50) and abstract-related words (*M* = 351, *SD* = 48, *r* = 0.95) *t*(17) = 1.6, *p* = 0.12, Hedges’ *g* = 0.11, *M*_diff_ = 6, 95% CI [-2; 13]; or between the RTs to leg/foot-related verbs (*M* = 355, *SD* = 53) and abstract-related words (*r* = 0.95), *t*(17) = -1.06, *p* = 0.31, Hedges’ *g* = 0.08, *M*_diff_ = 4, 95% CI [-13; 4] (**Table 3** and **Figure 4A**). No other simple effects were significant after controlling for multiple comparisons. Importantly, when analyzing hand and foot related words (ignoring abstract words) there was no hint of an interaction between Group and Verb, *F*(1,34) = 0.08, *p* = 0.781, ω_p_^2^ = -0.03, 95% CI [-0.03; 0.08].

### Color Discrimination Task: Movement Times

The same analysis of the MTs in the color discrimination task revealed a main effect of SOA, with faster responses on short SOA trials (*M* = 338, *SD* = 75) than on long SOA trials (*M* = 346, *SD* = 72), *F*(1,34) = 6.25, *p* = 0.017, *M*_diff_ = 7, 95% CI [1; 13], and a three-way interaction between Group, verb category, and SOA, *F*(2,68) = 4.32, *p* = 0.017, ω_p_^2^ = 0.09, 95% CI [-0.03; 0.22], but neither of these effects were significant after the Bonferroni correction. Importantly, the interaction between group and verb type was not statistically significant, *F*(2,68) = 0.87, *p* = 0.425, ω_p_^2^ = 0.00, 95% CI [-0.03; 0.09] (**Figure 4B**). When analyzing only hand and foot related words (ignoring abstract words) there was no interaction between Group and Verb, *F*(1,34) = 1.25, *p* = 0.272, ω_p_^2^ = 0.01, 95% CI [-0.03; 0.21].

### Color Discrimination Task: Errors

A three-way repeated-measures ANOVA [between-subjects factor: Group (DCD, TD); within-subjects factor: verb category (arm/hand-related, leg/foot-related, abstract-related) and SOA (short [53.2 ms], long [332.5 ms]) did not reveal any statistically significant effects (**Figure 4C**). Similarly, when analyzing only hand and foot related words (ignoring abstract words) there was no interaction between Group and Verb, *F*(1,34) = 0.10, *p* = 0.748, ω_p_^2^ = -0.03, 95% CI [-0.03; 0.13].

### Correlations between Size of the Interference Effect and the Scores of Clinical Scales

**FIGURE 5:**
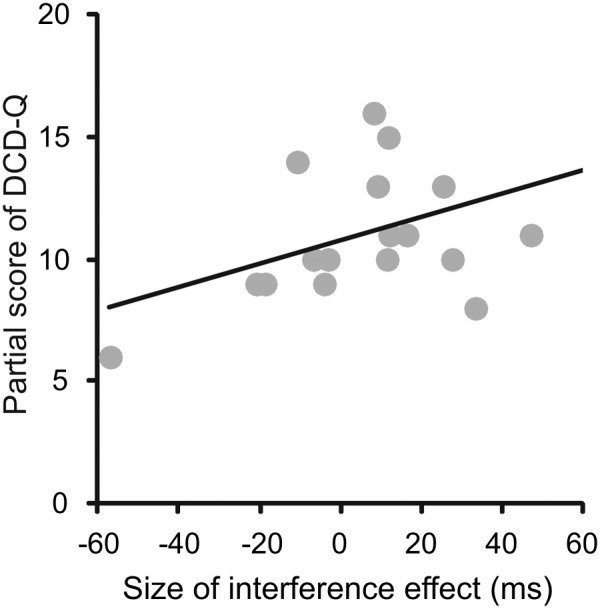
**Correlations between the size of the interference effect assessed from RTs and the partial score of the DCD questionnaires (DCD-Q) related to fine hand movements.** The size of the interference effect was always measured as the difference between the RTs of reaching arm movements after reading an arm/hand-related verb and the RTs of the same movements after reading a leg/foot-related verb. Even though all possible correlations were computed, here are shown just the one that was significant, i.e., the correlation between the partial scores of the DCD-Q related to fine hand movements and the size of the interference effect measured at the short SOA.

In order to see whether the seriousness of the disease was linked to the amount of the interference effect, we explored their correlation. To this end, we computed the Pearson’s correlations (*r*) between total and partial scores of the M-ABC and DCD questionnaires of each patient and the corresponding size of the interference effect (measured as the difference between the RTs of reaching arm movements after reading an arm/hand-related verb and the RTs of the same movements after reading a leg/foot-related verb) at the short SOA, at the long SOA and at the mean effect across these two values. The results indicated that there was a reasonably strong correlation between the partial score of the sub-scale of DCD questionnaire related to fine hand movements and the interference effect at the short SOA (*r* = 0.48, *p* = 0.044). Importantly, as negative values of the interference effect (i.e., when the RTs of reaches to leg/foot-related verbs were longer than those to arm/foot-related verbs) correspond to low values of the DCD scores (**Figure 5**), this correlation implies that those patients with a more severe form of the disease showed also a reversed interference effect. Although this result should be interpreted tentatively due to the small sample size and multiple comparisons that were not controlled for, this correlation deserve attention in future research (for all computed Pearson’s correlation values, see **Table 4**).

**Table 4:** Values of the Pearson’s correlations between total and partial scores of the Movement Assessment Battery for Children (M-ABC) and of the DCD-questionnaire (DCD-Q) and the corresponding size of the interference effect (IE) at the short SOA, at the long SOA and at the mean effect across these two values.

	IE short SOA	IE long SOA	IE mean across SOAs
DCD-Q control during movement	0.024	0.074	0.064
DCD-Q fine hand movements	0.480*	-0.019	0.398
DCD-Q general coordination	0.069	0.118	0.128
DCD-Q total score	0.239	0.094	0.259
M-ABC manual dexterity	0.206	-0.298	0.000
M-ABC ball skills	0.208	0.227	0.331
M-ABC static equilibrium	-0.171	0.186	-0.036
M-ABC dynamic equilibrium	-0.184	-0.091	-0.211
M-ABC total score	0.047	-0.026	0.025


*A star indicates a significant correlation at *p* < 0.05. See text for further details.*

## Discussion

The aim of the current study was to examine the hypothesis that action-verb processing is, at least partially, dependent on the motor system. Our results show a clear link between action verb understanding and a non-pathological functioning of the motor system, suggesting that the comprehension of verbal descriptions of actions at least partially relies on an internal simulation of the sensory-motor experience of the described action. We found that only TD children, and not those affected by DCD, exhibit an interference effect. When the cue to execute a reaching arm movement toward a peripheral target is a verb describing an action executed with the same effector used to give the motor response, only TD children showed an increase in RTs compared to when the cue is a verb describing an action involving another effector (the foot/leg, Mirabella et al., 2012; Spadacenta et al., 2014). Crucially, this phenomenon occurred only when the semantic content of a verb has to be retrieved, which suggests that in TD children the motor system is recruited if a verb must be understood, but not when other features of the word (i.e., its printed color) represent the rule to generate the appropriate response. However, in DCD children the RTs of movements triggered by arm/hand-related verbs did not differ from those triggered by leg/foot-related verbs, neither in the semantic task nor in the color discrimination task.

It must be stressed that the overall amplitude of the interference effect is relatively small, being about 20 ms. Thus, we believe that the phenomenon we observed represents a cost linked to the way in which the neural network subserving action-language processing is organized. *Per se*, in the real world, the interference effect does not provide advantages nor disadvantages, otherwise it would compromise our ability to react efficiently in presence of action-language material. Therefore, the lack of the interference effect in the DCD children cannot be though as an advantage, but it simply reflects a different degree of involvement of the motor system during processing action words in these patients with respect to healthy subjects.

In agreement with Mirabella et al. (2012), we found that the interference did not involve the MTs. Our analyses were motivated by the fact that on the one hand reaching movements are not ballistic and thus, in principle the MTs can be modulated according to the context at play (e.g., Mirabella et al., 2008, 2013). On the other hand, Desai et al. (2015) showed that implicit, but not explicit semantic action verb processing are related to the MTs. All in all, we confirmed that when the semantic content of a verb has to be explicitly retrieved, MTs are not affected. It is very well known that RTs and MTs reflect different stages of reaching movement, and it might be that only neural processes occurring during movement planning can be affected by semantic processing of action words, while those occurring during movement execution are not affected.

### Neural Basis of the Interference Effect

Our evidence is in line with other findings suggesting that in DCD patients the integrity of the motor network is disrupted. For instance, a recent diffusion tensor imaging study has reported reduced white matter integrity within the corticospinal tract in DCD patients (Zwicker et al., 2012). It has also been shown that cerebellar-parietal and cerebellar-prefrontal networks activity associated with skilled motor practice was under-activated in DCD patients with respect to TD children (Zwicker et al., 2011). Finally, a resting-state fMRI study revealed reductions in functional connectivity between the primary motor cortex and several other brain regions linked to processing of the motor output (McLeod et al., 2014). These neural damages could well account for the typical motor symptomatology characterizing this disease.

The correlation between the size of the interference effect with the score of the sub-scale of the DCD questionnaire related to the fine motor abilities, although tentative, suggests a possible link between the severity of the disease and a decreased ability to grasp the meaning of a verb to execute an action. Importantly, because our sample of DCD patients was not affected by specific learning disorders (which is a common comorbidity in this clinical population, for a review Visser, 2003), their impairment in the semantic task performance cannot be explained by a general linguistic disability. Conversely, it seems to be a genuine effect of motor impairments characterizing the DCD.

Supporting evidence to this hypothesis comes from a number of studies suggesting that DCD is related to a deficit of action representation rather than to an inability to produce the motor acts. On the one hand, it has been shown that children with DCD tend to be impaired on both gestures produced in the presence (transitive action) or in the absence (intransitive action) of an object during imitation and on verbal command with respect to TD children (Zoia et al., 2002; Goyen et al., 2011; Sinani et al., 2011). On the other hand, it has also been showed that DCD patients have motor imagery deficits (Williams et al., 2013). Motor imagery is an active cognitive process, which involves the internal re-enactment of a motor action without any overt motor output. According to the embodied theory of language such an impairment would affect action language understanding, which is exactly what we found.

This is not the first time that damage to the motor system has been found to be associated with a specific linguistic impairment. Some clinical studies have shown action verb deficits relative to concrete nouns in stroke patients with lesions relatively circumscribed to the motor regions (Neininger and Pulvermuller, 2003), as well as in patients with motor neuron disease (e.g., Bak et al., 2001; Grossman et al., 2008; Bak and Chandran, 2012) and Parkinson’s disease (Signorini and Volpato, 2006; Boulenger et al., 2008). However, these studies suffer from linguistic caveats, related to linguistic differences (e.g., word frequency, syntactic function, imageability, age of acquisition, etc.) between verbs and nouns. Two recent studies, one involving Parkinson’s patients (Fernandino et al., 2013) the other involving left stroke patients (Desai et al., 2015), represent a remarkable exception in that the psycholinguistic variables are carefully controlled and they both show to a causal relationship between sensory–motor and conceptual systems. Nevertheless, in both studies the cohort of patients are extremely heterogeneous. For instance, in the cohort of Fernandino et al. (2013) are included Parkinson’s patients with very different degree of disease severity (ranging from 1 to 4 of the Hoehn-Yahr scale). Furthermore, most of them had been tested after they took their dopaminergic therapy, but some were in OFF-therapy. Similarly, in Desai et al. (2015) the sites of the brain lesions of left stroke patients are not defined (about 74% are aphasic). Clearly these biases limit the interpretation of the results.

In addition, recently, the interpretation of some of the above mentioned studies (Bak et al., 2001; Bak and Chandran, 2012) has been questioned. Papeo et al. (2015) tested patients with amyotrophic lateral sclerosis and found that they do not suffer from a specific deficit in the processing of action-related verbs, but from a frontal-executive dysfunction which disrupts the mental representation of actions. In fact, Papeo et al. (2015) showed that when matching verbs and object nouns (in order to control for their semantic relationship with the same motor representations, e.g., ‘write’ and ‘pen’) the differences in performance between these two words categories still persisted.

Other clinical evidence sustaining the causal link between language processing and the motor systems comes from studies on Parkinson’s patients (e.g., Cardona et al., 2014; Melloni et al., 2015) exploiting the so-called action-sentence compatibility effect (ACE, Glenberg and Kaschak, 2002). This effect shows that comprehending a sentence describing an action denoting movements toward or away from the body (e.g., ‘Launch the ball’ or ‘Close the drawer’) facilitates movements in the same direction, but slows down movements in the opposite direction. Cardona et al. (2014) showed that ACE was impaired in early Parkinson’s patients. However, sentences have a high morphological and syntactic complexity, which might represent potential confounding variables in the interpretation of the results. In fact, in the same study Cardona et al. (2014) found that in patients with motor neuron disease the ACE effect was of a similar size as in healthy controls.

In conclusion, these confounds do not allow previous studies to provide unambiguous support for a role of motor systems in semantic language processing. However, these caveats do not apply to the present study, as we compared the same verbs under two different conditions in two well defined populations. In the semantic task, participants had to retrieve the semantic meaning of the verb to respond correctly, while in the color discrimination task, participants just needed to process the color of the printed verbs and not their meaning. Very likely, even in the latter task, participants might read the verbs, but they do not need to use the semantics to give the correct response. This suggests that under these circumstances the semantic representation of the action, at least partially stored in the motor cortex, does not have to be activated (Mirabella et al., 2012; Spadacenta et al., 2014). Thus, in our view, the difference in performance between DCD patients and TD children cannot be attributed to anything else than the motor disease.

Because there is no specific locus of damage within the motor system in DCD, we cannot speculate which part of the motor system is involved in language processing in the current study. Nor, we can exclude the possibility that the interference effect could be due to problems with the interaction between the motor system and other language-related brain regions. In fact, it is very likely that the semantic meaning of verbs is processed in a distributed network of brain regions to which both motor and non-motor regions contribute. In this regard, it has been proposed that children usually learn action words while performing actions, while they frequently hear the name of the executed action (usually a verb) from their parents or caretakers. The nearly simultaneous activation of motor and language brain regions would allow for a link to form between the neural representation of a word and the corresponding motor program (Pulvermuller, 2005). In adulthood these links can even change as a result of experience, as shown by the discovery that players of a given sport exhibit an improved comprehension of sport-specific action-related language (Beilock et al., 2008). This finding offers an explanation for another result in the present dataset. As opposed to young adults (about 26 years old, Mirabella et al., 2012; Spadacenta et al., 2014), neither DCD nor TD children showed a higher percentage of mistakes in the semantic task when they moved after reading an arm/hand-related verb compared to after reading a leg/foot-related verb. This phenomenon might suggest that at about 10 years of age the associative process between motor and language brain regions could be partially incomplete. In addition, the fact that overall DCD patients made more errors than TD children in the semantic task, but not in the color task, seems to suggest that when DCD children are performing a task where they need to associate the meaning of a verb to an action, they are generally more impaired than when they have to associate another cue (the color of the printed verb) to an action.

## Conclusion

All in all, our results support the notion that motor system plays a key role in action-language understanding in agreement with the embodied theories of language (Gallese and Lakoff, 2005; Vigliocco et al., 2009). Future studies will be needed in order to precisely define what regions of the motor system are related to action semantics processing.

## Author Contributions

GM designed the experiments, programmed the stimuli display, performed part of the analyses, produced the figures and wrote the manuscript. SDS selected, recruited patients, and collected experimental data. DL performed the statistical analyses and revised the manuscript. RA selected, recruited patients, and collected experimental data. RP selected, recruited patients, and revised the manuscript. FC selected, recruited patients, and revised the manuscript.

## Conflict of Interest Statement

The authors declare that the research was conducted in the absence of any commercial or financial relationships that could be construed as a potential conflict of interest.
